# A temporary cholesterol-rich diet and bacterial extracellular matrix factors favor *Salmonella* spp. biofilm formation in the cecum

**DOI:** 10.1128/mbio.03242-24

**Published:** 2024-12-05

**Authors:** Alonso Cruz-Cruz, Megan E. Schreeg, John S. Gunn

**Affiliations:** 1 Center for Microbial Pathogenesis, Abigail Wexner Research Institute at Nationwide Children’s Hospital, Columbus, Ohio, USA; 2 Department of Veterinary Biosciences, The Ohio State University, Columbus, Ohio, USA; 3 Infectious Diseases Institute, The Ohio State University, Columbus, Ohio, USA; University of Washington, Seattle, Washington, USA

**Keywords:** chronic carrier, lithogenic diet, cecum, biofilms, *Salmonella*

## Abstract

Asymptomatic chronic carriers occur in approximately 5% of humans infected with *Salmonella enterica* serovar Typhi (*S*. Typhi) and represent a critical reservoir for bacterial dissemination. While chronic carriage primarily occurs in the gallbladder (GB) through biofilms on gallstones, additional anatomic sites have been suggested that could also harbor *Salmonella. S*. Typhimurium, orally infected 129 × 1/SvJ mice were pre-treated with a cholesterol-rich diet as a gallstone model for chronic carriage. We observed *S*. Typhimurium in feces and the cecum during early and persistent infection. Furthermore, bacterial biofilm-like aggregates were associated with the cecum epithelium at 7 and 21 days post-infection (DPI) in mice on a lithogenic diet (Ld) and correlated with an increase in cecal cholesterol at 21 DPI. *Salmonella*’s extracellular matrix (ECM) was demonstrated as important in colonizing the cecum, as survival and aggregate formation significantly decreased when mice were infected with a quadruple ECM mutant strain. Gallbladder *Salmonella* counts were low at 36 DPI while cecal *Salmonella* were high, suggesting that gallbladder colonization was likely not responsible for the high cecal burden. All cecum phenotypes were significantly diminished in mice fed a normal diet (Nd). Finally, we examined the capability of *S*. Typhi to colonize the cecum and showed *S*. Typhi in feces and in aggregates in the cecum up to 7 DPI, with slightly higher counts in mice fed a Ld compared to Nd. Our findings suggest that the cecum, particularly under cholesterol-rich conditions, serves as an adaptative niche for *Salmonella* spp. aggregates/biofilms and is a putative site for long-term infection.

**IMPORTANCE:**

Typhoid fever is a systemic infectious disease triggered by the gastrointestinal dissemination of *Salmonella* Typhi and Paratyphi in humans. Three to five percent of infected individuals become chronic carriers, a state in which gallstone biofilm formation facilitates spread of the bacteria in feces. Notably, surgical removal of the gallbladder (GB) in some chronic carriers (22%) does not guarantee the elimination of the bacteria, and the rationale for this remains poorly understood. This study is significant as it explores other tissues associated with the chronic carrier state. It highlights not only a cholesterol-rich diet as an important etiological factor for *Salmonella* colonization but also identifies the cecum as a crucial tissue promoting fecal shedding. Additionally, we determined that biofilm matrix components of *Salmonella* are key factors contributing to these effects. A greater understanding of these mechanisms will allow the formulation of new therapeutic strategies specifically targeted at preventing typhoid fever dissemination from chronic carriers.

## INTRODUCTION

*Salmonella* Typhi (*S*. Typhi) is responsible for causing millions of infections annually, particularly in endemic regions such as Asia and Africa. Infection by *S*. Typhi or *Salmonella* Paratyphi can lead to the development of typhoid fever, a systemic disease that affects the bloodstream, the gastrointestinal tract, and organs rich in macrophages (1). This infection gives rise to severe complications that are compounded by the emergence of multidrug-resistant (MDR) strains (2), representing a significant public health challenge. Antibiotic use has enabled effective treatment for *S*. Typhi infections in humans, allowing control of the infection in most patients. Nonetheless, a small percentage of patients (3%–5%) are unable to completely clear the infection and become chronic carriers. In these carriers, *Salmonella* persists for an extended period (at least 6 months) after the initial exposure (3). Although these carriers do not exhibit signs, they can excrete bacteria in their stool, representing a potential source of infection (4).

Prior research from our lab and others has highlighted the significance of the gallbladder (GB) in humans for the long-term viability of the bacteria in carriers. Specifically, we demonstrated the formation of *S*. Typhi biofilms on gallstones of chronic carrier patients and *S*. Typhimurium (*S*. Tm) biofilms on gallstones in a lithogenic mouse model that promotes gallstone formation (5). Biofilms are complex communities of bacteria that adhere to surfaces and are encased in a self-produced extracellular matrix (ECM). In *S*. Tm, the ECM primarily includes DNA, cellulose, colanic acid, O-antigen capsule, and the amyloid curli fimbriae. Cell-autonomous polysaccharide components like O-antigen capsule and colanic acid are involved in capsule biosynthesis. These cell-specific structures play a key role in protecting the cell from environmental stress and determining its antigenic properties. Additionally, the ECM contains components such as DNA, cellulose, and curli fimbriae that benefit the biofilm community (6, 7). Consequently, the GB has been considered a key organ where *Salmonella* colonizes during persistent infection. In a previous study, we found the presence of live *S*. Tm in feces at 3, 6, 9, and 12 months after intraperitoneal (IP) infection. Interestingly, the bacteria were infrequently recovered in GB (8). Additionally, in a study of chronic carrier patients (*n* = 45) who underwent cholecystectomy (removal of the GB), 22.2% of these patients continued to exhibit active bacterial excretion, extending up to 6 months (9). These findings suggest that other anatomical sites might actively contribute as a source of *Salmonella* dissemination.

In this context, a study conducted by Miller et al. (10) demonstrated that in susceptible mice (C57BL/6) previously treated with antibiotics to reduce the microbiota and colonization resistance, *S*. Tm forms aggregates in the luminal space of both cecum and colon after an intragastric infection. Utilizing immunofluorescence (IF) microscopy, it was shown that *S*. Tm was associated with curli expression, one of the key components of the *Salmonella* biofilm ECM. Similarly, the presence of *S*. Tm was detected in the gastrointestinal tract through luminescence imaging in 129 × 1/SvJ mice during acute infection (10). However, it remains unclear whether this phenomenon is exclusive to the early stages of infection and its significance in bacterial fecal excretion. Additionally, it is uncertain whether these observations can be replicated in an infection model without alterations to the microbiota.

In this study, we aimed to evaluate the behavior of *S*. Tm during natural intragastric administration and elucidate its behavior in immunocompetent 129 × 1/SvJ mice subjected to a cholesterol-rich diet/lithogenic diet (Ld) or a normal diet (Nd). We also investigated the presence of *S*. Tm and *S*. Typhi biofilms in the cecum and their potential links to the chronic carrier state. Our findings reveal a compelling connection between *Salmonella* spp. aggregation, a lithogenic diet, and the cecum, which together might comprise an optimal host environment that triggers biofilm formation and fosters the cecal persistence of *Salmonella* during chronic infection.

## RESULTS

### Transitory lithogenic diet promotes cecal colonization and persistence after a natural intragastric infection

**Fig 1:**
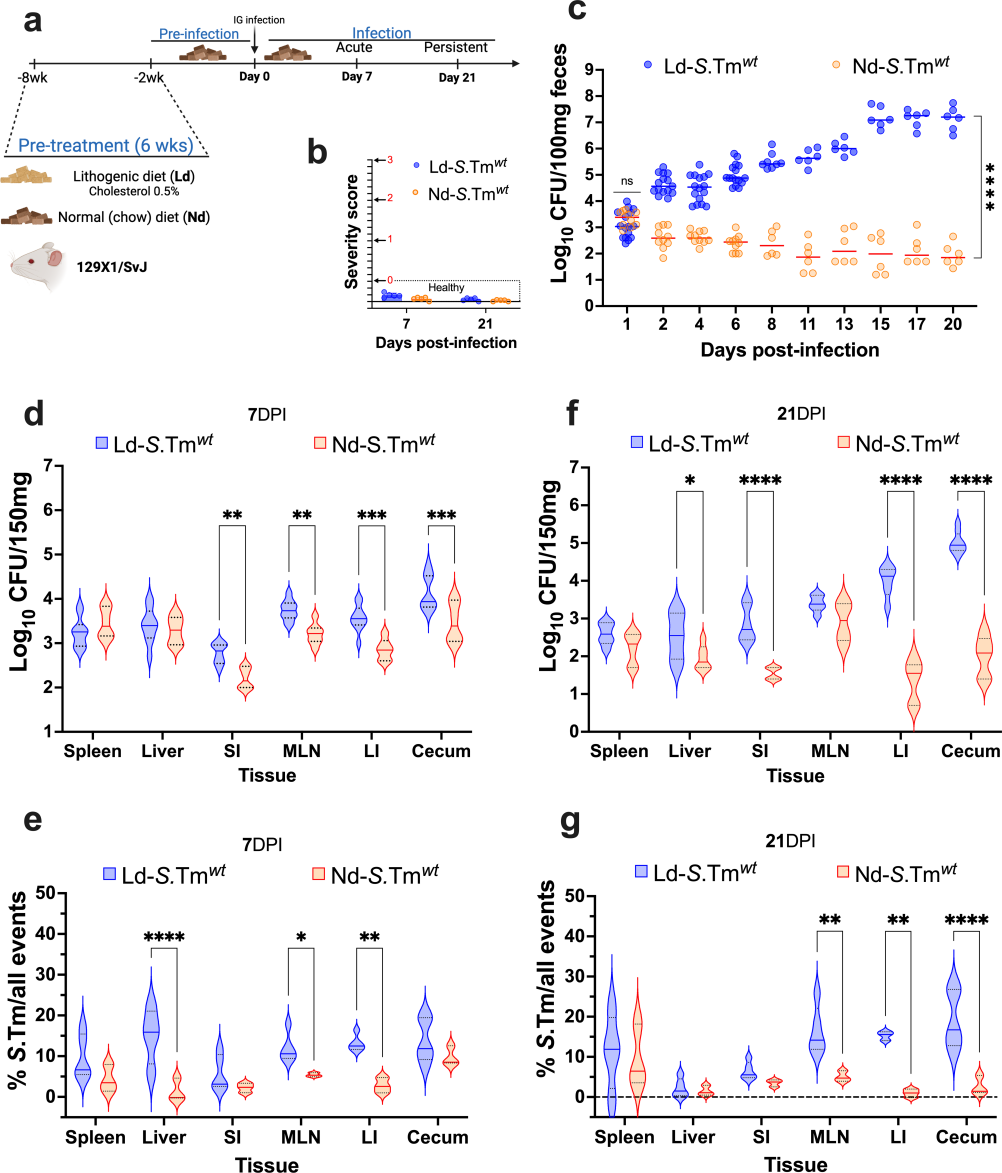
A lithogenic diet in mice promotes asymptomatic gastrointestinal colonization after intragastric infection with *S*. Tm*^wt^*. (**a**) Schematic representation of the experimental strategy. Mice (129 × 1/SvJ) were pre-treated with either a lithogenic (Ld) or a normal diet (Nd) for 6 weeks and were infected by the intragastric route with 1 × 10^7^ CFUs of *S*. Tm*^wt^*. Tissue samples were collected at 7 and 21 DPI. (**b**) Severity scores were assigned to both groups at specified times. The severity level is denoted with black arrows, a score of ≤0 representing a healthy animal. (**c**) Gastrointestinal tract colonization of mice was assessed by quantifying CFUs of freshy collected fecal samples. (**d**) Tissue samples were collected, homogenized, and either plated on selective XLD agar or (**e**) analyzed using flow cytometry at 7 DPI. MLN, mesenteric lymph nodes; SI, small intestine; and LI, large intestine. Similar quantification of *S*. Tm*^wt^* was performed at 21 DPI using (**f**) CFUs and (**g**) flow cytometry analysis. Flow cytometry analyses were normalized to a PBS control. At least six mice per condition were included. Dotted line represents ±quartiles. Statistical analysis was conducted using two-way ANOVA followed by Fisher’s least significant difference (LSD) test with significance levels indicated as follows: *P < 0.05, **P < 0.01, ***P < 0.001, and ****P < 0.0001.

Previously, we established a mouse *S*. Tm chronic carrier model by pre-treating mice with a Ld followed by IP infection (5). Here, we pre-treated 129 × 1/SvJ mice with either a transitory Ld or Nd for 6 weeks. Following this pre-treatment phase (both pre-infection and infection phases), all mice were subsequently fed a Nd. These mice were subjected to a single intragastric infection with 1 × 10^7^ colony forming units (CFU) of *S*. Tm wild type (*S*. Tm*^wt^*). Based on previous studies (11), we defined the early infection phase at 7 days post-infection (DPI) and the persistent infection phase at 21 DPI (Fig. 1a). Notably, throughout the infection phases, these mice did not display any clinical signs (Fig. 1b), indicating the potential establishment of an asymptomatic infection. Next, we evaluated the presence of *S*. Tm in fecal samples, using CFUs as a readout of colonization, and observed a progressive increase in the quantity of bacteria excreted over the course of the infection in mice subject to a Ld. Conversely, in feces of mice fed a Nd, we observed a decrease in bacterial fecal shedding over time (Fig. 1c). Intriguingly, we also observed a gradual reduction in the abundance of non-*Salmonella* gut microbiota, particularly the genus *Escherichia coli*, within the feces of mice subjected to the Ld (data not shown). This phenomenon was not observed in the fecal samples from mice on Nd. These observations suggest that dietary cholesterol might significantly influence *Salmonella*’s ability to colonize the gastrointestinal tract.

Furthermore, we explored the presence of *S*. Tm in various tissues following gastrointestinal infection. During early stages of the infection, we observed the survival of *S*. Tm in tissues including the spleen, liver, mesenteric lymph nodes (MLN), large intestine, small intestine (SI), and cecum in both groups of mice. A slight increase was observed in mice fed a Ld. We confirmed these findings through CFU counts (Fig. 1d) and flow cytometry analysis (Fig. 1e). Remarkably, at 21 DPI, we noted a significant increase in bacterial load in the large intestine and cecum of mice on Ld, demonstrated by both CFUs (Fig. 1f) and flow cytometry (Fig. 1g). These observations emphasize the crucial role of dietary factors not only in the initial establishment of *S*. Tm colonization but also in its capacity to adapt to specific tissue environments during infection.

### The *S*. Tm individual and combined ECM mutants demonstrate increased fecal and organ loads when mice are fed a Ld

In human hosts, a crucial mechanism for persistent survival in the gastrointestinal tract involves the formation of biofilms. A recently human study showed a remarkably high rate of bacterial biofilm formation in the cecum, which remains consistent whether individuals are in pathological or healthy conditions (12). To explore the role of *Salmonella* ECM components *in vivo*, we employed single mutants including curli fimbriae (*ΔcsgA*), colanic acid (*ΔwcaM*), O-antigen capsule (*ΔyihO*), and cellulose (*ΔbcsE*). None of these mutants displayed a growth deficiency *in vitro* compared to WT strain (Fig. S1a and b). Also, mice infected with these mutants did not display any clinical signs of infection (data not shown). When mice on a Ld were infected with these single mutants, we observed a progressive increase in bacterial counts in their feces versus those mice on a Nd, which significantly decreased (Fig. S2a). Next, we examined the bacterial burdens in the tissues at 21 DPI. Strikingly, there was a pronounced bacterial load in the gastrointestinal tract of mice fed a Ld but not a Nd (Fig. S2b). Thus, individual ECM mutants did not demonstrate a reduced ability to colonize the gastrointestinal tract and organs in mice fed a Ld, showing similarity to *S*. Tm*^wt^* infection.

Recognizing the collective role of self (capsule) and community (biofilm) factors responsible for biofilm formation and their potential implications in persistent infection, we conducted an experiment involving the infection of mice with a quadruple mutant strain (*ΔwcaM, ΔcsgA, ΔyihO,* and *ΔbcsE*; referred to as ECM*^mut^*). We found that the ECM*^mut^* strain survived in feces throughout all infection phases, following the behavior of single ECM mutants with respect to dietary conditions (Fig. S2a). Likewise, we observed similar characteristics to single ECM mutants in the ECM*^mut^* strain when exploring the gastrointestinal tract and tissue colonization at 7 and 21 DPI, determining a higher adaptability of the bacteria in mice pre-treated with a cholesterol-rich diet (Fig. S2b and c).

### *S*. Tm ECM promotes long-term survival in cecum

**Fig 2:**
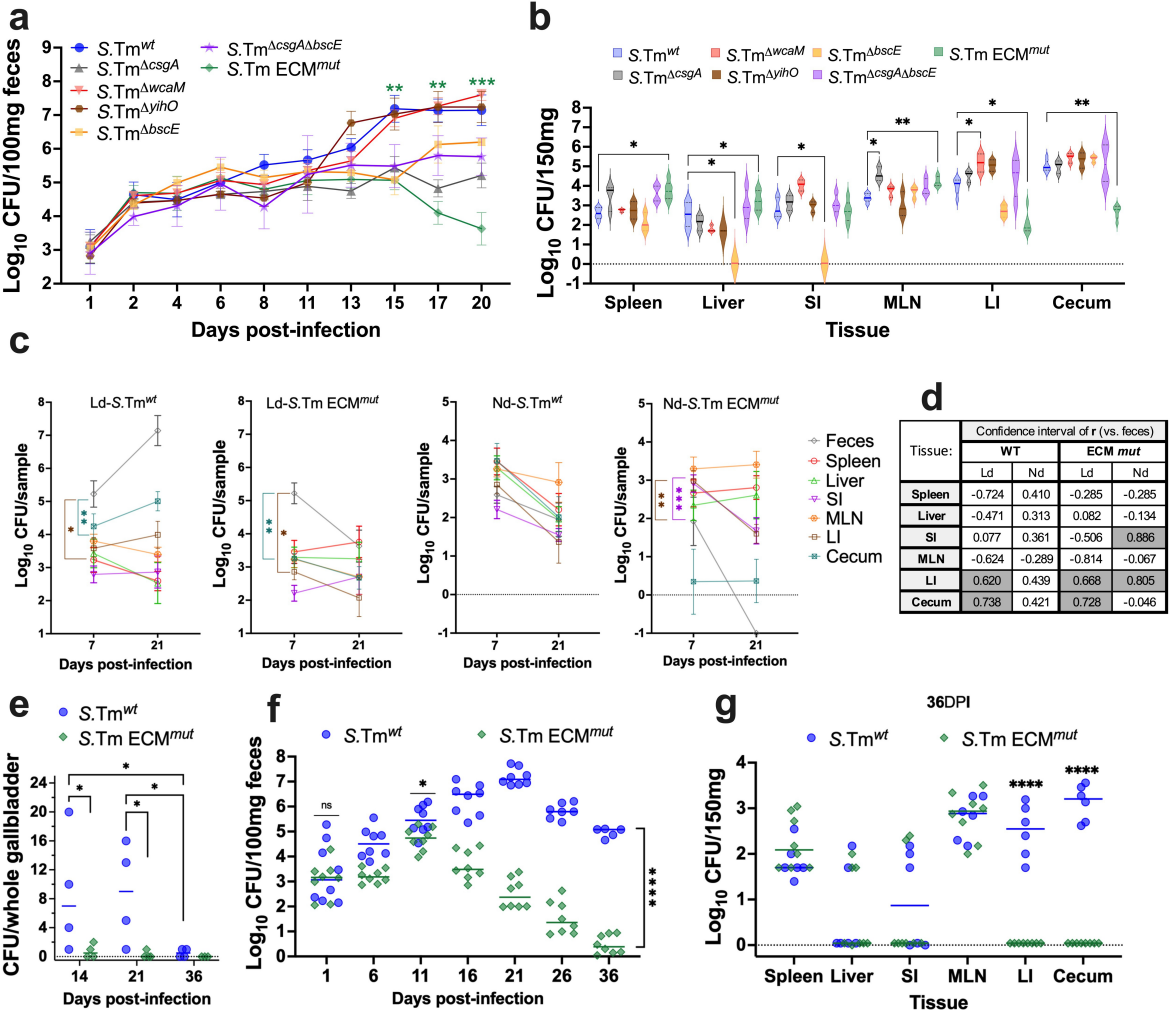
*S.* Tm ECM and the cecum are important components to establish a long-term infection. Mice fed with a Ld were infected with *S*. Tm ECM single mutants, each lacking curli (*ΔcsgA*), colanic acid (*ΔwcaM*), O-antigen capsule (*ΔyihO*), cellulose (*ΔbcsE*) genes, or a quadruple mutant lacking in all the above (ECM*^mut^*). (**a**) Bacterial burden was analyzed to quantify CFUs of freshly collected fecal samples. Strains are indicated. (**b**) CFUs were determined by culturing tissue samples on selective agar. MLN, mesenteric lymph nodes; SI, small intestine; and LI, large intestine. (**c**) Pearson correlation analysis was conducted between CFUs obtained from feces and tissues at 7 and 21 DPI. (**d**) *R* values from Pearson’s correlation analysis are presented in the table. Tissue, diet, and strain are indicated. (**e**) CFUs from the whole GB isolated from infected mice on a Ld. (**f**) Quantification of bacteria from freshy collected feces of infected mice on a Ld. (**g**) Bacterial burden in tissues of infected mice on a Ld at 36 DPI. A minimum of four mice per condition was utilized for these analyses. Dotted line represents ±quartiles. Error bars represent ±SD. Statistical analysis was conducted using two-way ANOVA followed by Fisher’s LSD test. *P < 0.05, **P < 0.01, ***P < 0.001, and ****P < 0.0001.

During the early phase of infection (<8 DPI), the number of bacteria in the feces of mice on Ld infected with single, double biofilm mutant (*S*. Tm^*Δ*^*^csgA^*^*Δ*^*^bcsE^*) or quadruple ECM*^mut^* mutants resembled the bacterial loads observed in *S*. Tm*^wt^*-infected mice. However, we noted a gradual decrease in fecal bacterial load in the capsule-/biofilm-deficient ECM*^mut^* strain after 8 DPI, highlighting the importance of both components for colonization (Fig. 2a). Subsequently, we conducted an analysis of tissue samples using CFUs. The ECM*^mut^* strain exhibited tropism for the spleen, liver, and MLN. The cellulose mutant showed a significant decrease in colonization of the liver and SI, but not other tissues. In contrast, with the *S*. Tm*^wt^*, the ECM*^mut^* strain (but not single or double mutants) exhibited a pronounced progressive deficiency in colonization in both large intestine and cecum (Fig. 2b). These collective findings underscore the critical role of the combined ECM components in both fecal shedding and gastrointestinal colonization.

Previous studies have alluded to the simultaneous presence of a considerable number of bacteria in both the cecum and feces following an intragastric and IP infection, suggesting a crucial role of the cecum in maintaining *Salmonella* excretion in feces, although this association has been poorly explored (13, 14). To investigate this, we studied the correlation between CFUs in feces and tissues at 7 and 21 DPI. Remarkably, a robust positive correlation in *S*. Tm*^wt^* was identified in the cecum (*r* > 0.7) and large intestine (*r* > 0.6) from mice on Ld, whereas this correlation was absent in mice on a Nd. Notably, no significant correlation was found with other tissues (Fig. 2c). Conversely, a negative correlation was observed in the cecum (*r* > 0.7) and large intestine (*r* > 0.6) from ECM*^mut^*-infected mice fed with a Ld. This correlation was even more pronounced (*r* > 0.8) in small/large intestine of these mice with Nd (Fig. 2d). In summary, these results not only imply a robust connection between the cecum and *Salmonella* excretion in feces during long-term infections but also emphasize the influence of the Ld and ECM components in shaping cecum colonization.

### Bacterial long-term shedding in feces is independent of the bacterial load in the GB

The GB has been commonly associated with the chronic carrier state. While most patients who undergo cholecystectomy manage to clear the infection, the role of this tissue in an oral infection in animal models has been poorly explored. Despite establishing a clear association between bacterial shedding and the bacteria present in the cecum and large intestine, we investigated the potential role of the GB in maintaining bacterial shedding in our model. To achieve this, mice on a Ld were infected with *S*. Tm*^wt^* and ECM*^mut^*, monitoring the bacterial counts in the GB, feces, and tissues. We found a higher bacterial count in the GB of mice infected with the *S*. Tm*^wt^* compared to those infected with the ECM*^mut^* at 14 and 21 DPI, but not at 36 DPI, where they were both low and near the detection limit (Fig. 2e).

Surprisingly, we detected bacterial shedding at 36 DPI in *S*. Tm*^wt^*-infected mice, which was significantly lower in mice infected with the ECM*^mut^* (Fig. 2f). When we analyzed the bacterial load in the tissues at 36 DPI, we did not detect differences in bacterial counts in the tissues except the cecum and large intestine, where the CFUs of mice infected with *S*. Tm*^wt^* was significantly higher than in mice infected with the ECM*^mut^* (Fig. 2g). These findings not only confirm the role of the ECM genes and the cecum in promoting long-term infection, but also suggest that the bacterial burden in the cecum and large intestine is independent of the GB.

### Biofilm components promote an immune response producing morphological changes in the cecum

**Fig 3:**
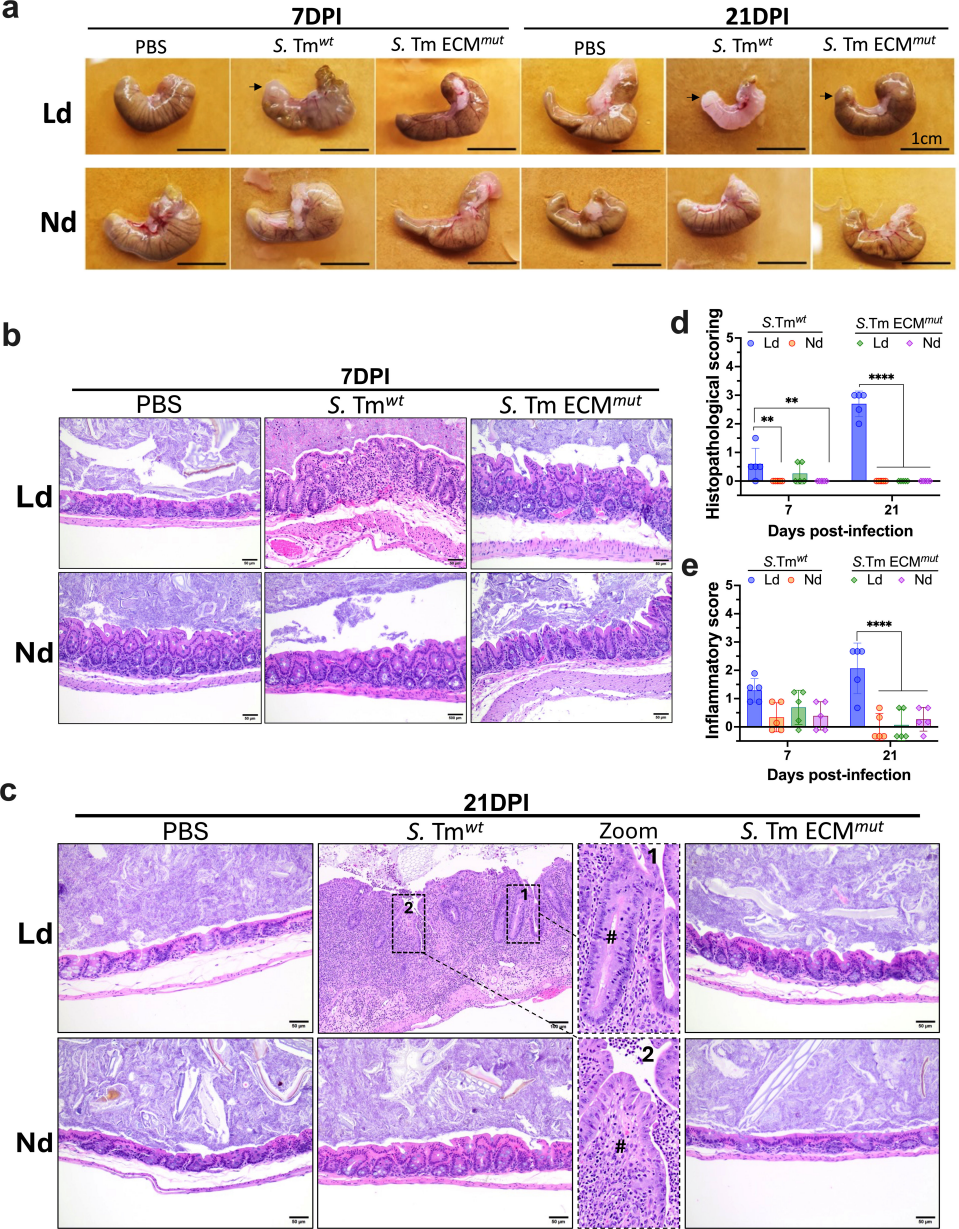
Immune response associated with the ECM components induces morphological changes in the cecum during persistent infection. (**a**) Anatomic and morphological changes in the cecum of infected mice were examined. Diet and strains are indicated. Cecal patches are highlighted by black arrows. (**b and c**) H&E staining of histological sections of the cecum. Day, diet, and strains are indicated. Optical magnification 40×, scale bar: 50 µm. Zoom image: crypt hyperplasia (#) and inflammatory cells are denoted in 1. Ulceration (#) and neutrophilic inflammation are denoted in 2. Optical magnification 20×, scale bar: 100 µm. (**d**) Histopathological and (**e**) inflammatory scores were evaluated from these sections at 7 and 21 DPI. The scores were normalized to the PBS control. Error bars represent ±SD. At least four mice per condition were included in this study. Statistical analysis was conducted using two-way ANOVA followed by Fisher’s LSD test. **P < 0.01 and ****P < 0.0001.

We examined the macroscopic morphology of the cecum at 7 and 21 DPI. Anatomic morphological changes were observed at 21 DPI on *S*. Tm*^wt^*-infected mice with Ld. The cecum showed reduced size and an apparent high grade of fibrosis. Importantly, these changes did not manifest in the cecum of mice infected with the ECM*^mut^* strain or any other conditions (Fig. 3a).

Previous *in vivo* studies have demonstrated a robust association between infection and the immune response in the cecum. However, these models often rely on the use of streptomycin to eliminate the microbiota (15, 16). To delve more deeply into our anatomical observations, we explored histopathological changes including hyperplasia, inflammation, fibrosis, and crypt modification of the cecum. We observed a significant intraluminal and intraepithelial immune infiltration at 7 DPI in the cecum of mice infected with *S*. Tm*^wt^* on a Ld but not a Nd (Fig. 3b and c). These features intensified during the persistent infection, accompanied by hyperplasia, fibrosis, goblet cell loss, crypt distention, and ulceration. These modifications were in line with histopathology (Fig. 3d) and inflammatory scores (Fig. 3e). Remarkably, all these alterations were absent in mice infected with *S*. Tm ECM*^mut^*. This indicates that ECM components possess significant immunogenicity and aid colonization, leading to the recruitment of immune system components that induce substantial anatomical changes in the cecum in the presence of a Ld.

### Cholesterol is localized to the cecum in Ld-fed mice weeks after the diet is stopped

**Fig 4:**
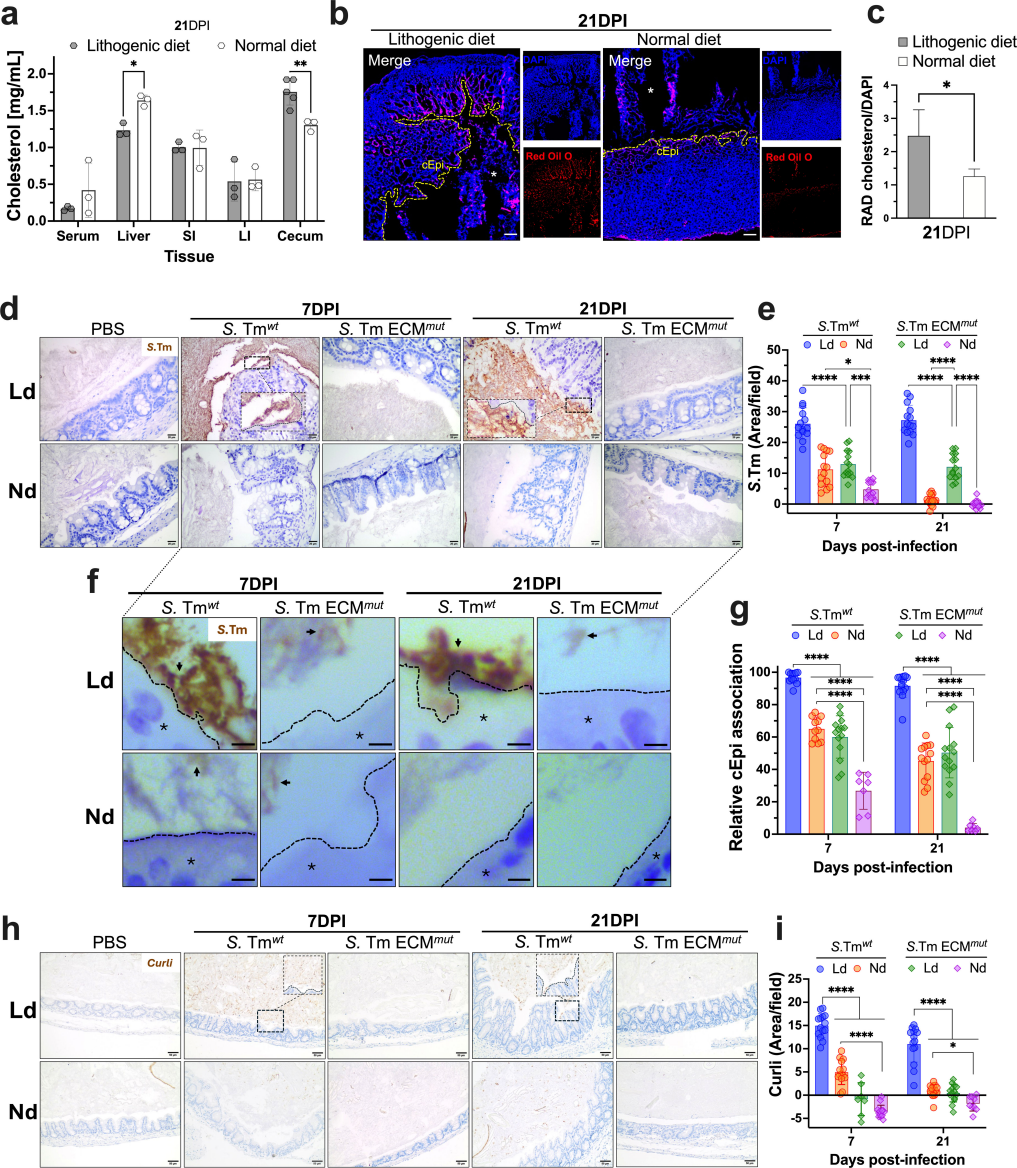
Close association between *S*. Tm and the cecal epithelium is related to cholesterol presence. (**a**) Cholesterol concentrations were determined in homogenized tissues at 21 DPI. SI, small intestine; LI, large intestine. (**b**) Frozen samples were isolated at 21 DPI from *S*. Tm*^wt^*-infected mice. Staining was performed using Red Oil O (red) to identify cholesterol and DAPI (blue) as counterstain. cEpi, cecal epithelium. Optical magnification 20×, scale bar: 100 µm. The asterisks represent the cecal lumen. (**c**) Quantification of Red Oil O signal was normalized with DAPI. RAD, relative abundance distribution. (**d**) Distribution of *S*. Tm in the cecum samples from infected mice using IHC. Optical magnification 40×, scale bar: 20 µm. (**e**) Quantification of *S*. Tm positive signal in panel d. Strains and dietary conditions are indicated. (**f**) Visual association between *S*. Tm and cEpi was carried out using IHC. The black dashed lines delineate the cEpi border, and the asterisks indicate the cEpi. The black arrows show a positive signal for *S*. Tm. Optical magnification 60×, scale bar: 5 µm. Primary Ab *Salmonella* CSA-1 was employed in panels d and f. (**g**) Quantification of relative association between *S*. Tm and the cEpi. (**h**) IHC of *S*. Tm curli expression in the cecum. Strains and dietary conditions are indicated. Optical magnification of 20×, scale bar: 50 µm. (**i**) Quantification of the curli positive signal. In all cases, at least five images per cecum were captured using light microscopy. All IHC quantifications were normalized to the PBS control and quantified using ImageJ. Error bars represent ±SD. Dotted lines represent ±quartiles in panel c. At least four mice per condition were utilized. Statistical analysis was conducted using two-way ANOVA followed by Fisher’s LSD test. *P < 0.05, ***P < 0.001, and ****P < 0.0001.

Considering the significant role of the Ld in our mouse model, we conducted further experiments to assess cholesterol concentrations across all tissues. We observed a notable increase in cholesterol concentration at 21 DPI within the cecum of mice previously fed with a Ld compared to a Nd (Fig. 4a; Fig. S3b). We did not find any significant differences in cholesterol concentrations between the infected and non-infected mice. This suggests that the presence of *S*. Tm in the cecum may not significantly alter the distribution of cholesterol in this tissue. Also, we found that cholesterol was readily distributed in the cecal epithelium and lumen, supporting its potential importance and role during the development of biofilms in both short- and long-term infection (Fig. 4b). In addition, we found a relative higher cholesterol abundance using IF microscopy (Fig. 4c). This variation in cholesterol levels could potentially contribute to the tissue-specific adaptation of the *Salmonella* and its biofilm-forming capacity.

### *S*. Tm association with the cecal epithelium is linked with biofilm formation

Given the recognized critical role of the ECM in the *Salmonella*’s persistence within the cecum, we explored biofilm formation in this anatomical region, assessing *S*. Tm localization through immunohistochemistry (IHC). Our results confirmed a robust association between *S*. Tm*^wt^* and both the cecal epithelium and the lumen at 7 and 21 DPI. Intriguingly, this intimate association was predominantly observed in *S*. Tm*^wt^*-infected mice on a Ld, while reduction and redistribution of the bacteria were noted in samples from mice on a Nd. Notably, *S*. Tm*^wt^* was more abundant than the ECM*^mut^* strain in both diet conditions (Fig. 4d). To further confirm these findings, we quantified the positive chromogenic signal, consistently supporting our visual observations (Fig. 4e). Importantly, these results held true when using an alternative specific antibody against *S*. Tm (Fig. S2c).

Furthermore, we sought to gauge the proximity of *S*. Tm to the cecal epithelium by measuring the area between the apical face of the cecal epithelium and the *S*. Tm signal within the cecal lumen. This measurement was inversely correlated with the degree of epithelial association where a close physical connection between *S*. Tm*^wt^* and the cecal epithelium was observed in Ld-fed infected mice. This association was substantially disrupted in both diet groups infected with the *S*. Tm ECM*^mut^* strain and samples from infected mice with *S*. Tm*^wt^* on Nd (Fig. 4f and g). To support the biofilm presence, curli was also detected in association with the cecal epithelium under similar dietary and strains conditions (Fig. 4h and i).

### *S*. Tm forms biofilms in the cecum of mice fed a Ld

**Fig 5:**
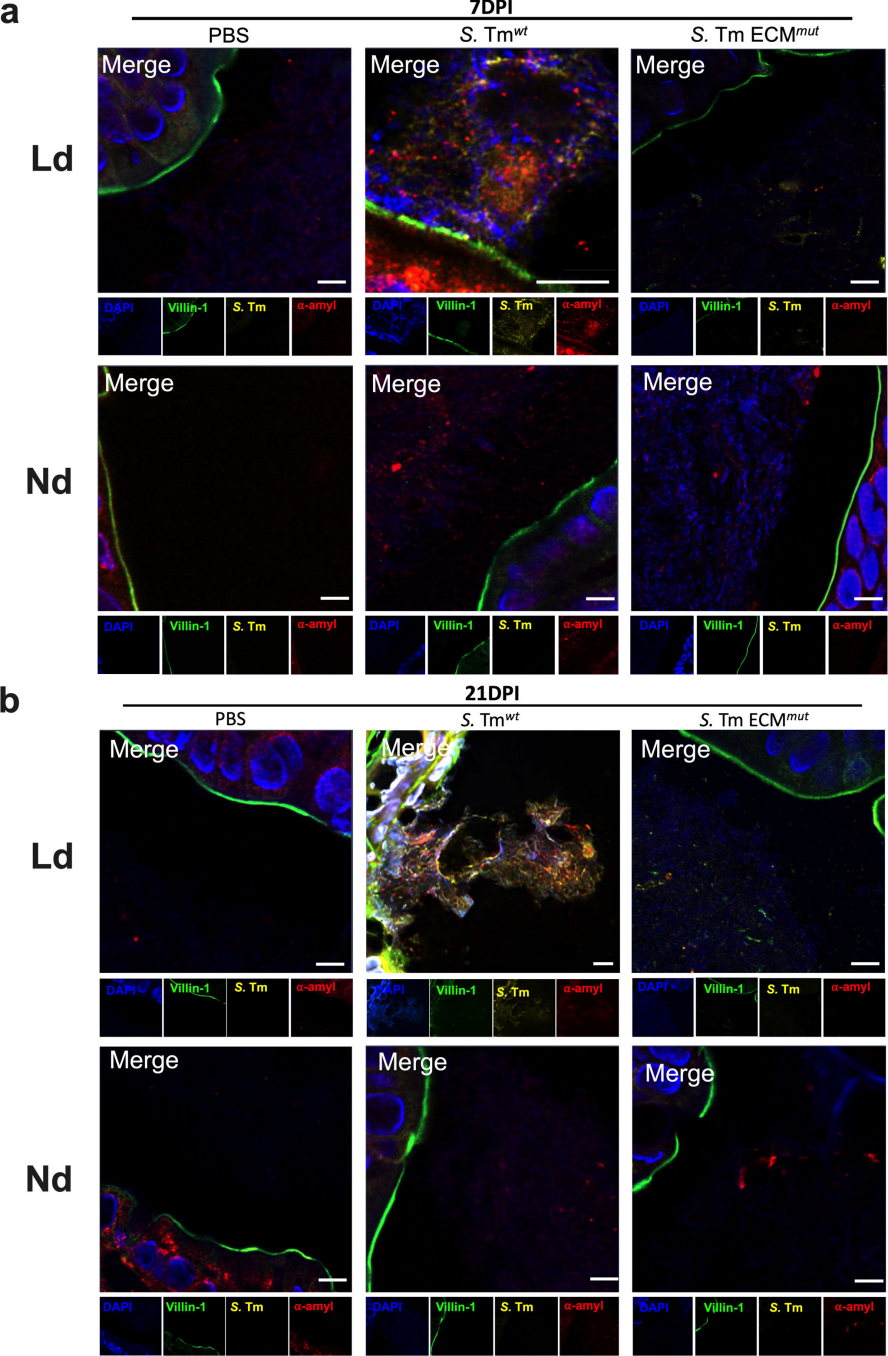
*S.* Tm forms biofilms in cecum during both short- and long-term infection. IF images displaying the visualization of biofilms on cecum samples obtained from mice infected with *S*. Tm*^wt^* or *S*. Tm ECM*^mut^* at (**a**) 7 or (**b**) 21 DPI. Dietary conditions are indicated. Biofilms were detected using red for alpha-amyloid protein (curli), yellow for a *S*. Tm (CSA-1), green for villin-1 (a cecal epithelium marker), and blue for DNA (DAPI). Optical magnification 60×, scale bar: 5 µm. At least six mice per condition were utilized for this analysis.

Given the lack of biofilms formed in the absence of ECM components, we next examined *Salmonella* ECM biofilm factors including curli (amyloid), cellulose, and DNA. We detected *S*. Tm associated with the cecal epithelium, exhibiting co-localization with DNA and alpha-amyloid proteins exclusively in the *S*. Tm*^wt^* at 7 and 21 DPI only when mice were fed a Ld (Fig. 5a and b). Furthermore, we validated this association with cellulose and lipopolysaccharide (LPS) on sequential histological cuts at 7 DPI (Fig. S3d). Collectively, these findings strongly underscore the significance of *Salmonella* biofilms in sustaining *S*. Tm persistence within the cecum. Moreover, they emphasize the crucial role of the ECM as a vital component for colonizing the cecum and facilitating the formation of biofilms.

### *S*. Typhi forms biofilms in the cecum of mice fed a Ld

**Fig 6:**
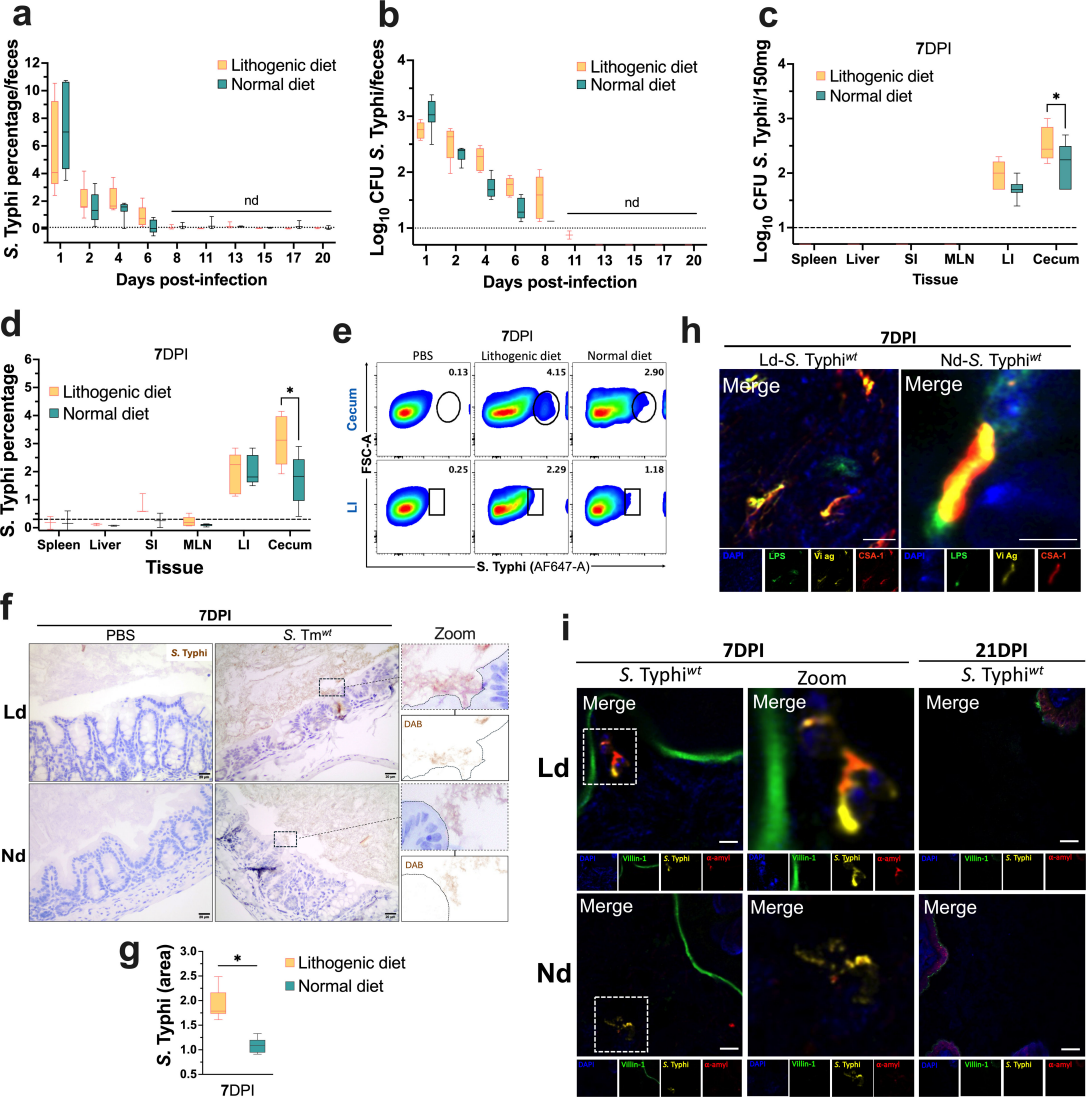
*S.* Typhi forms biofilms in cecum from mice fed with a high-cholesterol diet (Ld). Mice fed with either Ld or Nd were orally administered with 5 × 107 CFUs of *S*. Typhi WT *rpoS^+^* (*S*. Typhi*^wt^*). The extent of colonization in mouse tissues was assessed using (**a**) CFUs and (**b**) flow cytometry. Tissue homogenates were isolated at 7 DPI for quantification via (**c**) CFUs and (**d**) flow cytometry. MLN, mesenteric lymph nodes; SI, small intestine; and LI, large intestine. (**e**) Dot plots illustrate the identification of *S*. Typhi’s population in the cecum and LI at 7 DPI. (**f**) IHC images visually represent the presence of *S*. Typhi*^wt^* in the cecum of infected mice at 7 DPI. Dietary conditions are indicated. Primary Ab CSA-1 was employed. Optical magnification 40×, scale bar: 20 µm. (**g**) Quantification of chromogenic signal in panel f. (**h**) IF to identify *S*. Typhi*^wt^* in the cecal lumen. Dietary conditions are indicated. Staining was performed using α-Vi Antigen (ViAg), α-*S*. Typhi LPS (LPS), and α-CSA-1 (*S*. Typhi). Optical magnification 60×, scale bar: 2.5 µm. (**i**) *S*. Typhi biofilms in the cecum at 7 and 21 DPI. Biofilms were detected using the same protocol for *S*. Tm in Fig. 5. Optical magnification 60×, scale bar: 5 µm. The flow cytometry and quantification data were normalized to the PBS control in all cases. All IHC quantifications were quantified using ImageJ. At least five mice per condition were utilized. Error bars represent ±SD, and dotted lines present ±quartiles. Two-way ANOVA and Fisher’s LSD test were used (a to d), and Student’s *t*-test was performed in panel f. *P < 0.05.

*S*. Typhi, confined to human hosts, has lacked a suitable animal model for the investigation of its pathophysiology (17). Here, we aimed to explore the capacity of *S*. Typhi *rpoS^+^* (*S*. Typhi*^wt^*) to persist within and form biofilms in the cecum. Mice on Ld and Nd were infected intragastrically with 5 × 10^7^ CFUs of *S*. Typhi*^wt^*. As expected, no clinical signs of infection were observed in the mice (data not shown). A transient colonization of the gastrointestinal tract was noted, which diminished after 7 DPI in both groups (Fig. 6a and b). Viable bacteria were detected in the large intestine and cecum at 7 DPI under both dietary conditions as evidenced by CFUs (Fig. 6c) and flow cytometry (Fig. 6d and e), with a slight increase in the cecum of mice fed a Ld. The presence of bacteria was not identified in any other tissues examined. The specificity of these colonies was confirmed by flow cytometry (Fig. S3e). We also performed morphological studies, which showed no significant anatomical or histopathological alterations in the cecum of these mice (Fig. S3f). IHC analysis revealed the association of *S*. Typhi with the cecal epithelium, showing a slight increase in mice fed a Ld (Fig. 6f and g). Furthermore, IF microscopy provided evidence of *S*. Typhi in the cecal lumen in both dietary groups through targeting various *S*. Typhi antigens including the Vi antigen, LPS, and common structural antigen 1 (CSA-1) (Fig. 6h). Finally, *S*. Typhi was found to be associated with cecal epithelium in mice fed a Ld, but not under a Nd, where the bacteria were predominantly confined to the lumen. This association was accompanied by the presence of DNA and alpha-amyloid protein, suggesting the biofilm formation under lithogenic conditions (Fig. 6i). Together, these results underscore the significant impact of diet on *Salmonella* spp. biofilm formation, even in a host that is not optimal for *S*. Typhi infection.

**Fig 7:**
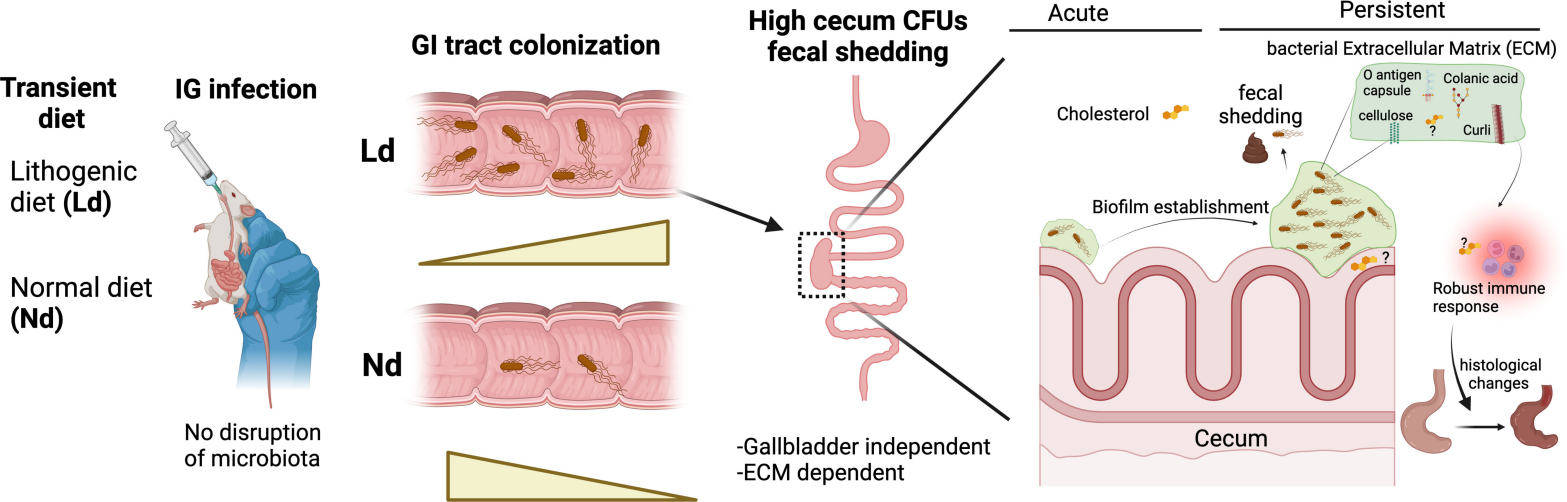
Graphical summary. *Salmonella* oral infection in immunocompetent mice pre-treated with a Ld elicits a robust immune recruitment in the cecum, requiring the presence of ECM components. The ECM components are associated with cecal biofilm formation during early and long-term infection contributing to *Salmonella* persistence in this tissue. IG, intragastric; GI, gastrointestinal.

In summary, our findings collectively underscore *Salmonella*’s remarkable adaptability to form biofilms within the cecum during both early and persistent infections, supported by a robust recruitment of immune system components. These characteristics work synergistically to ensure its survival and the subsequent establishment of persistence following a natural route of infection (Fig. 7).

## DISCUSSION

The chronic carrier state is a significant occurrence in typhoid fever patients worldwide, with carriers playing a crucial role in the spread of typhoidal *Salmonella* infection (3). Therefore, it is vital to explore all potential factors involved in the development and maintenance of the chronic carrier state. In this study, we developed a mouse intragastric infection model to simulate a human chronic carrier state to investigate the importance of diet and biofilm formation during this process. Our data demonstrate that a transient cholesterol-rich diet significantly increases *Salmonella* colonization in the gastrointestinal tract, particularly the large intestine and the cecum. Moreover, we found that ECM components and cholesterol are indispensable for biofilm formation in the cecum, thus promoting the establishment of persistent infection.

Previous studies have emphasized the significance of the spleen, GB, and mesenteric lymph nodes as significant reservoirs for *Salmonella* during long-term infections. These studies have suggested several mechanisms including granulomas, phenotypic variation biofilms or intracellular localization within macrophages (5, 18–20). Recent research in humans has shown that the cecum, in both healthy and pathological conditions, exhibits a strikingly higher percentage (70%–90%) of bacteria forming biofilms compared to other segments of the gastrointestinal tract. In our chronic carrier model, we observed a robust association between *Salmonella* spp. and the cecum, leading us to designate the cecum as an additional important tissue involved in bacterial shedding and dispersion (Fig. 2i and j).

Other research groups have reported the formation of *Salmonella* biofilms in the colon and cecal lumen of microbiota-depleted mice following intragastric infection (10, 12). In our current study, we demonstrated the presence of *Salmonella* spp. biofilms in the cecum without disrupting the microbiota (Fig. 4c and 5). Additionally, we confirmed the significance of the ECM in the development of chronic carriage, as the *S*. Tm*^wt^* strain, but not the quadruple ECM mutant, was found in the cecum in mice fed a Ld and induced a robust and sustained immune response (Fig. 4). ECM is essential for robust biofilm formation but also affects the immune response against the bacterium (18). These observations align with prior reports in diverse infection models, particularly during the early stages of infection (15, 16, 18). Notably, the curli fimbriae has been characterized as a potent immunogen, especially when in association with DNA, another ECM component. This combination has been found to trigger proinflammatory immune responses in macrophages (21). Here, we observed the importance of ECM antigens especially during persistent infection and colonization (>8 DPI) (Fig. 2a). These findings collectively emphasize the complexity of the immune response triggered by *Salmonella* infection, and underscore the roles played by various components and dietary factors in shaping these responses, especially within the context of intestinal inflammation (22–24).

The role of cholesterol in *Salmonella*’s adaptation, survival, and pathogenicity has been explored by *in vitro* assays (25–28). We noticed higher cholesterol concentrations in the cecum of mice fed a Ld compared with those fed a Nd, even weeks after the Ld was stopped. Furthermore, the bacterial numbers in the cecum were dramatically higher than those in the GB at 36 DPI, reinforcing the long-term cecal adaptation of *Salmonella*, during this dietary conditions (Fig. 2e through g). In these conditions, cholesterol appeared to be predominantly associated with the lumen and cecal epithelium, and was linked to enhanced *Salmonella* survival and biofilm formation, as depicted in Fig. 4. In this context, we have previously shown that *Salmonella* can bind to cholesterol to initiate biofilm formation, where flagellar mutants were unable to initiate cholesterol binding (29). In this context, no significant differences were observed in curli promotor activity when incubated with cholesterol versus without cholesterol, either at 26°C (Fig. S4a) or at 37°C (Fig. S4b). This suggests that there is no direct relationship between cholesterol and the expression of biofilm-associated genes. On the other hand, host cholesterol has been recognized as an essential carbon source that supports the survival and persistence of *Mycobacterium* tuberculosis during chronic infections (30). However, whether *Salmonella* exhibits similar characteristics in this context remains uncertain. Alternatively, a recent study suggested that a cholesterol-rich diet does not significantly impact the composition of the gut microbiota, which is a crucial factor in resisting *S*. Tm colonization in mice (31). Controversially, other studies suggest that a short-term high-fat diet rich in oleic acid enhances *S*. Tm gastrointestinal colonization while decreasing *E. coli* resistance in susceptible mice (C57BL/6) over a brief period. However, microbiota colonization resistance was restored with 48 hours after returning to a normal diet. In contrast, our results show a high level of colonization when Ld-fed mice were infected, highlighting the distinct role of cholesterol compared to other types of fats (32).

Following the *S*. Tm studies, we explored into the adaptability of *S*. Typhi in this model, which is typically rapidly eliminated in a mouse without causing signs of disease. In doing so, we not only observed an increased relative ability of *S*. Typhi to form aggregates and associate with the cecal epithelium, but also noted tissue-specific affinity. In this context, recent studies have described the presence of asymptomatic intestinal and fecal carriage for invasive non-typhoidal strains in individuals from endemic regions (33, 34). This suggests that our observations may represent a possible mechanism of asymptomatic gastrointestinal colonization for *Salmonella* spp. in humans.

In our research, we have demonstrated that a lithogenic diet increases *Salmonella* spp. colonization of the gastrointestinal tract. Moreover, we found that high cholesterol concentrations in the cecum were linked to *Salmonella* biofilm formation and likely persistent infection. We hypothesize that, like biofilms on gallstones, *Salmonella*’s predilection for cholesterol aids colonization, biofilm formation, and persistence. However, additional experiments are necessary to elucidate the precise role of cholesterol in this process.

## MATERIALS AND METHODS

### Bacterial strains

Previously, we described the construction of *Salmonella* mutant strains deficient in biofilm-formation capacity (6). The following bacterial strains were utilized in this study: *S*. Tm 14028 (*S*. Tm*^wt^*; JSG210), *S*. Tm curli mutant (*S*. Tm^*Δ*^*^csgA^*; JSG3540), *S*. Tm colanic acid mutant (*S*. Tm^*Δ*^*^wcaM^*; JSG3712)*, S*. Tm O-antigen capsule mutant (*S*. Tm^*Δ*^*^yihO^*; JSG3672), *S*. Tm cellulose mutant (*S*. Tm^*Δ*^*^bcsE^*; JSG3838), the double ECM mutant (*S*. Tm^*Δ*^*^csgA^*^*Δ*^*^bcsE^*; JSG3977), *S*. Tm quadruple ECM mutant *ΔwcaM, ΔcsgA, ΔyihO, ΔbcsE* (*S*. Tm ECM*^mut^*; JSG3841), *S*. Tm containing a curli gene promoter fusion to luciferase (*S*. Tm*^lux:csgDEFG^*; JSG3977), and *S*. Typhi *rpoS*^+^ Ty2 (*S*. Typhi*^wt^*; JSG698). Planktonic cultures were cultivated for 16 hours at 37°C in tryptic soy broth (TSB). All strains were regrown to an OD_600_ of 0.6 prior to infection. Mice were infected intragastrically with a dose of 1 × 10^7^ for *S*. Tm or 5 × 10^7^ for *S*. Typhi.

### *In vitro* bacterial growth curves

Overnight (O/N) cultures of *Salmonella* WT and mutant strains were normalized to an OD_600_ of 1. The cultures were then diluted 1:1,000 in fresh TSB medium and incubated at 37°C. Bacterial growth was monitored over a 15-hour period, with OD_600_ readings taken every 15 minutes.

### Luciferase assay

O/N cultures of *S*. Tm*^lux:csgDEFG^* were normalized to an OD_600_ of 1. The cultures were subsequently diluted 1:1,000 in fresh Luria-Bertani (LB) medium and incubated at either 26°C or 37°C in microtiter plates in the presence or absence of cholesterol (well coating). The strain was grown over a 15-hour period, with luminescence (OD_490_) readings taken every 15 minutes.

### Bacterial shedding and fecal collection

Fresh fecal samples were obtained by individually placing mice in isolation containers until approximately 100 mg of feces was collected. The collected fecal samples were homogenized in 1 mL of sterile phosphate-buffered saline (PBS) and plated on selective agar xylose lysine deoxycholate (XLD) (refer to “Bacterial Isolation” below), followed by incubation for 24 hours (*S*. Tm) or 48 hours (*S*. Typhi) at 37°C.

### Clinical assessment of mice

Mice were evaluated at 7 and 21 DPI to assess phenotypic signs of the severity of the infection. The evaluation included measurements of body weight, assessment of general health (fur, eyes, posture, clinic complications), and observations of motility.

### Mouse infection model and tissue CFUs

Six-week-old female 129 × 1/SvJ mice (The Jackson Laboratory, Maine, USA) were utilized in this study. Prior to infection, the mice were fed either a normal diet or a lithogenic diet for 6 weeks. The lithogenic diet consisted of mouse chow supplemented with 0.5% cholesterol and 0.5% cholic acid (Envigo, TD 140673). Two weeks after the diet treatment, the mice were intragastrically infected with *S*. Tm or *S*. Typhi in 200 µL of PBS containing the bacteria.

### CFUs from feces and tissues: bacterial isolation

Mice were euthanized at 7, 21 or 36 DPI. The liver, spleen, mesenteric lymph nodes, small intestine, large intestine, and cecum were isolated. Approximately 150 mg of tissues or feces was homogenized in 1 mL of sterile 1× PBS. The samples were briefly centrifuged for 30 seconds, and the supernatant was filtered through a 15-mm strainer. The filtrate was transferred to a U-bottom 96-well plate and subjected to centrifugation at 1,500 rpm for 8 minutes at 5°C. The supernatants were transferred to new wells (x3), followed by centrifugation at 3,700 rpm (8 minutes, 5°C) to obtain a bacterial pellet. The pellet was resuspended and plated on selective XLD agar to determine the bacterial load of each organ (Fig. S3a). The remaining tissues were preserved for cholesterol analysis, histology, or flow cytometry.

### Bacterial flow cytometry

Following the isolation of bacteria (as described above), the pellet was blocked with 5% bovine serum albumin (BSA) for 10 minutes and washed with 3% BSA in PBS at 3,700 rpm (8 minutes, 5°C). Primary antibodies (5 µg mL^−1^) and secondary antibodies (2.5 µg mL^−1^) were incubated with the pellet at 37°C for 20 minutes. Each antibody incubation was followed by three washes. The pellets were resuspended in 300 µL of PBS, the samples were analyzed using flow cytometry (BD LSRII), and the data were analyzed in FlowJo v10. The following antibodies were used: goat α-*S*. Tm/Typhi CSA-1 (BacTrace, 5310-0322), mouse IgG2a α-*S.* Typhi LPS (Meridian Bio, C01362M), rabbit IgG α-*S*. Tm (Thermo, PA-17244), α-mouse IgG2a FITC (Invitrogen, 11421082), and α-goat AF647 (Invitrogen, A21469) and 4′,6-diamidino-2-phenylindole (DAPI) (Invitrogen, D1306).

### Cholesterol quantification

After the initial centrifugation of bacteria at 3,700 rpm, 50 µL of supernatant from homogenized tissue was collected, and the cholesterol concentration was measured using the Amplex Red Cholesterol Assay Kit following the manufacturer’s instructions.

### Histology

All tissues were fixed in 10% neutral-buffered formalin (Fisher Scientific, Massachusetts, USA) for 24 hours. The morphology core at Abigail Wexner Research Institute embedded the tissues in paraffin. Deparaffinization and rehydration of samples were carried out before staining. Permeabilization was performed by incubating the samples in PBS containing 1% Triton X-100 for 1 hour at 37°C. Histological sections of 4 µm were used for hematoxylin and eosin (H&E) staining, IHC, or IF microscopy. For IHC and IF, the primary antibody (Ab) incubation was performed by overnight incubation at 4°C in antibody dilution buffer (3% BSA and 0.35% Triton X-100 in PBS) with 0.5–1 μg mL^−1^ for each Ab. The antibodies used included a goat IgG α-*S*. Tm/Typhi CSA-1, mouse IgG2a α-*S.* Typhi LPS, rabbit IgG α-*S*. Tm, rabbit α-Villin 1 (Abclonal, A11650), rabbit IgG α-Vi Antigen (Abcam, ab79002), Human α-amyloid APP 3H3 (Creative Bio, TAB-0801CLV), and pAb rabbit α-*S*. Tm CsgA (gift from Dr. Cagla Tükel). For IHC, after primary Ab incubation, the VECTA Elite ABC Kit-HRP (Vector Lab, PK-6101 and PK6105) and UltraTek Bio-HRP α-goat IgG (ScyTek Labs, ABL/AGL015) were used, and staining was revealed using the metal enhancer DAB Kit (Thermo, PI34065). The slides were then counterstained with hematoxylin for 30 seconds and washed in dH_2_O for 1 minute. Finally, coverslips were applied using ProLong mounting media (Thermo, P36930).

### Staining of frozen tissue sections

All tissues were fixed for 24 hours. Next, the tissue was embedded in Tissue-Tek and was frozen. Frozen tissue sections of 4 µm were prepared for IF staining. For identifying cholesterol, a Red Oil O stain kit (Abcam, ab150678) was used following the manufacturer’s instructions. DAPI was employed as a counterstain. The samples were quenched and mounted as described earlier. Images were acquired using a Zeiss Axiolab 5 microscope for H&E/IHC and Zeiss LSM 800 confocal microscope for IF. Fiji/ImageJ was utilized for image quantification, with the H DAB plugin to quantify the horseradish peroxidase (HRP) chromogenic signal and the wound healing assay plugin employed to analyze the area between bacteria and cecal epithelium (35, 36).

### Histologic evaluation and scoring of ceca

Cecal histology was evaluated by a board-certified veterinary anatomic pathologist who was blinded to study groups. Morphologic and inflammatory changes in tissues were scored using a modified scoring system that was based off of previously published diagnostic guidelines used in mice and dogs (37, 38); scores were tabulated, and morphologic diagnoses were assigned for each individual evaluated. Briefly, criteria evaluated included epithelial injury, crypt changes, presence of fibrosis, goblet cell loss, pseudo polyps, intra-epithelial inflammatory cells, inflammatory cell type, luminal inflammatory exudate, transmural inflammation, and the presence of lymphoid follicles.

### Statistical analysis and data graphics

Most of the data were evaluated and plotted in Prism version 9. For this study, the analysis was determined by two-way analysis of variance (ANOVA) or Student’s *t*-test. BioRender software was used to generate a representative illustration of the study (https://BioRender.com).

## SUPPLEMENTAL MATERIAL

Fig. S1 mbio.03242-24-s0001.tiff Bacterial growth curves.

Fig. S2 mbio.03242-24-s0002.tiff Gastrointestinal tract colonization.

Fig. S3 mbio.03242-24-s0003.tiff Methodology, quantifications, biofilms, and typhi data.

Fig. S4 mbio.03242-24-s0004.tiff Luciferase activity of the curli (csgDEFG) promoter.

Legends mbio.03242-24-s0005.docx Supplemental figure legends.

ASM does not own the copyrights to Supplemental Material that may be linked to, or accessed through, an article. The authors have granted ASM a non-exclusive, world-wide license to publish the Supplemental Material files. Please contact the corresponding author directly for reuse.
