# Supplementary figures and images for: A temporary cholesterol-rich diet and bacterial extracellular matrix factors favor Salmonella spp. biofilm formation in the cecum
Source: mBio. 2024 Dec 5;16(1):e03242-24. doi: 10.1128/mbio.03242-24 (PMC11708031; doi:10.1128/mbio.03242-24)

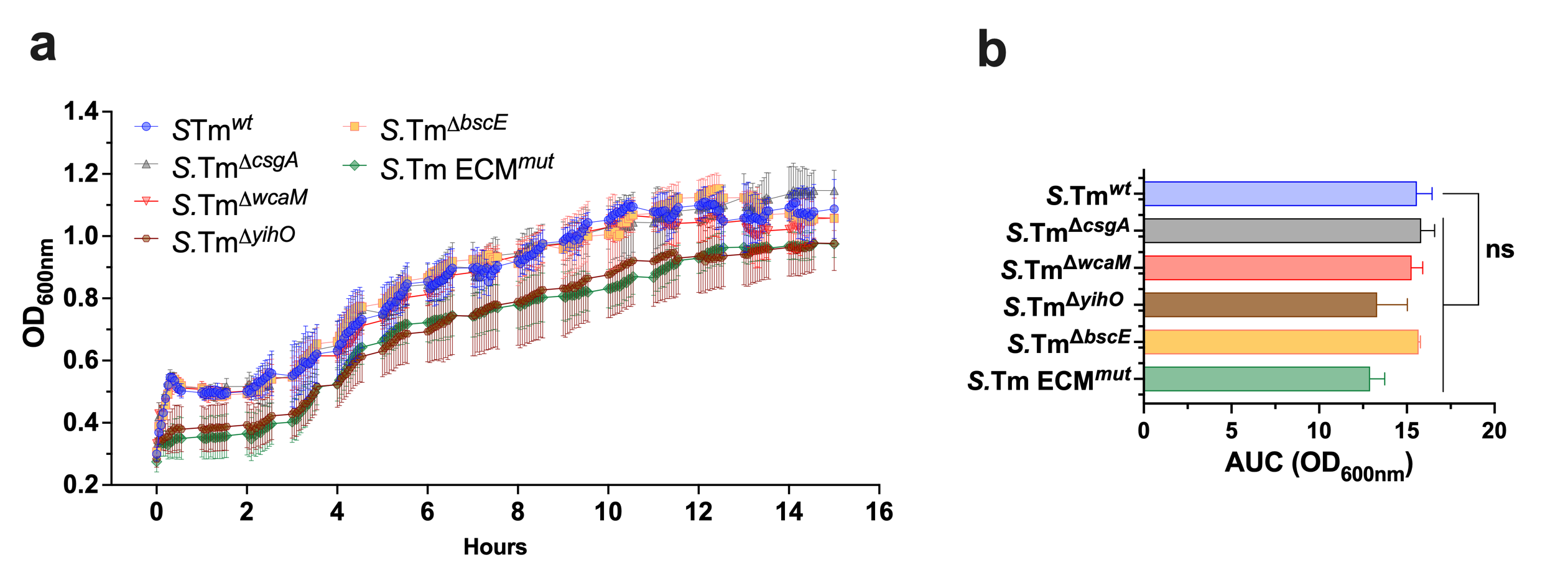

Supplement: Fig. S1 — Bacterial growth curves. [file mbio.03242-24-s0001.tiff]

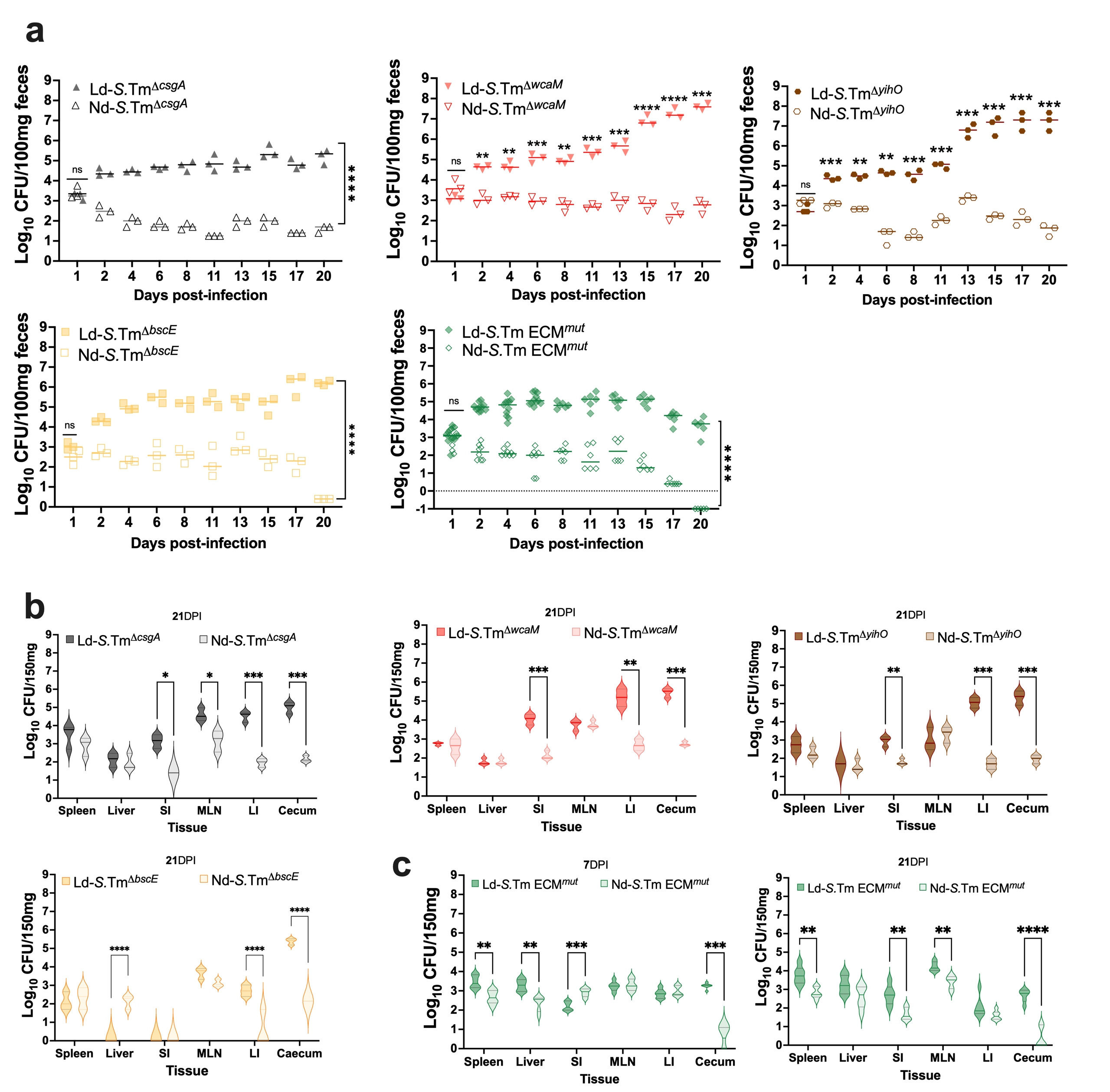

Supplement: Fig. S2 — Gastrointestinal tract colonization. [file mbio.03242-24-s0002.tiff]

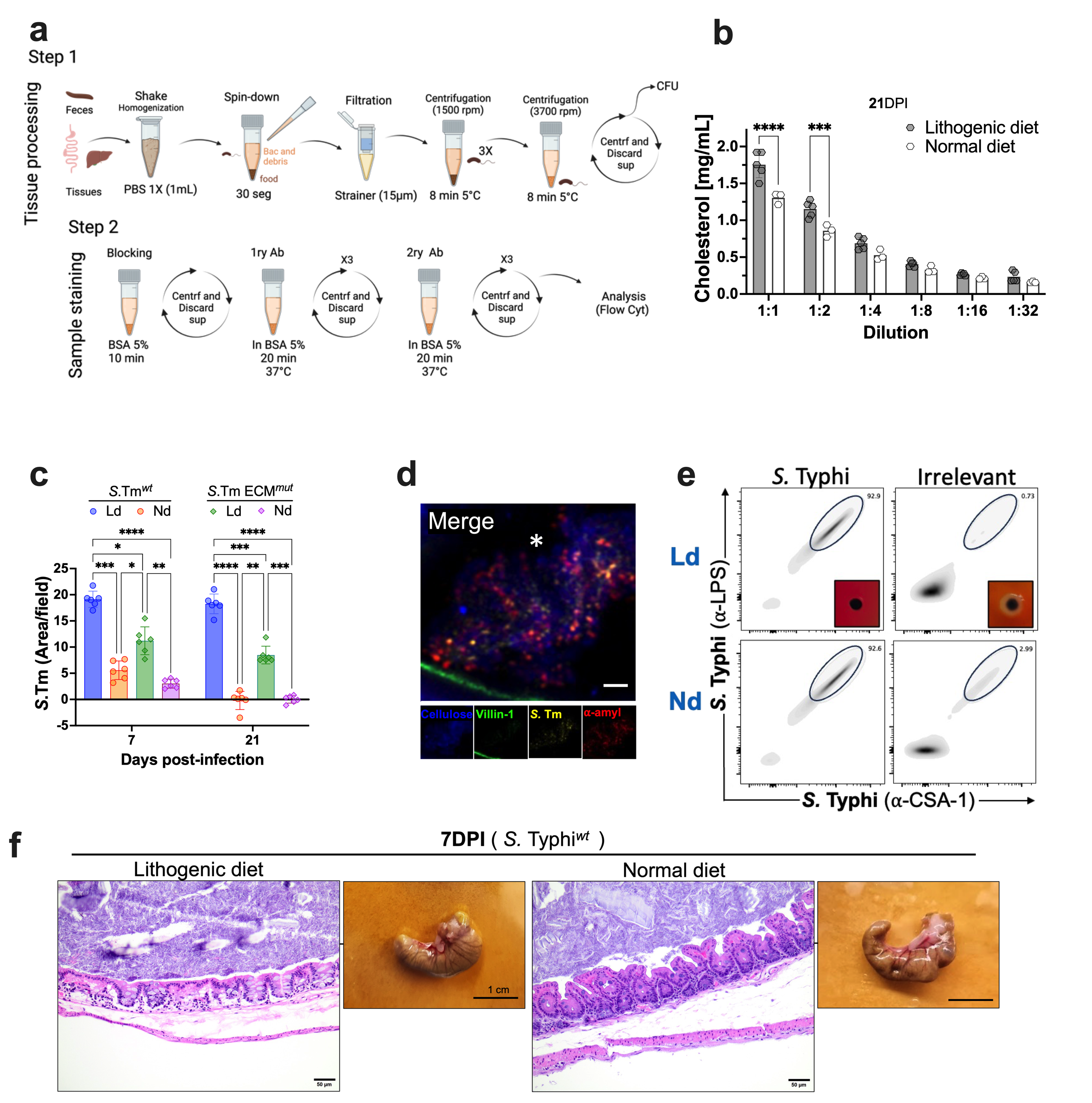

Supplement: Fig. S3 — Methodology, quantifications, biofilms, and typhi data. [file mbio.03242-24-s0003.tiff]

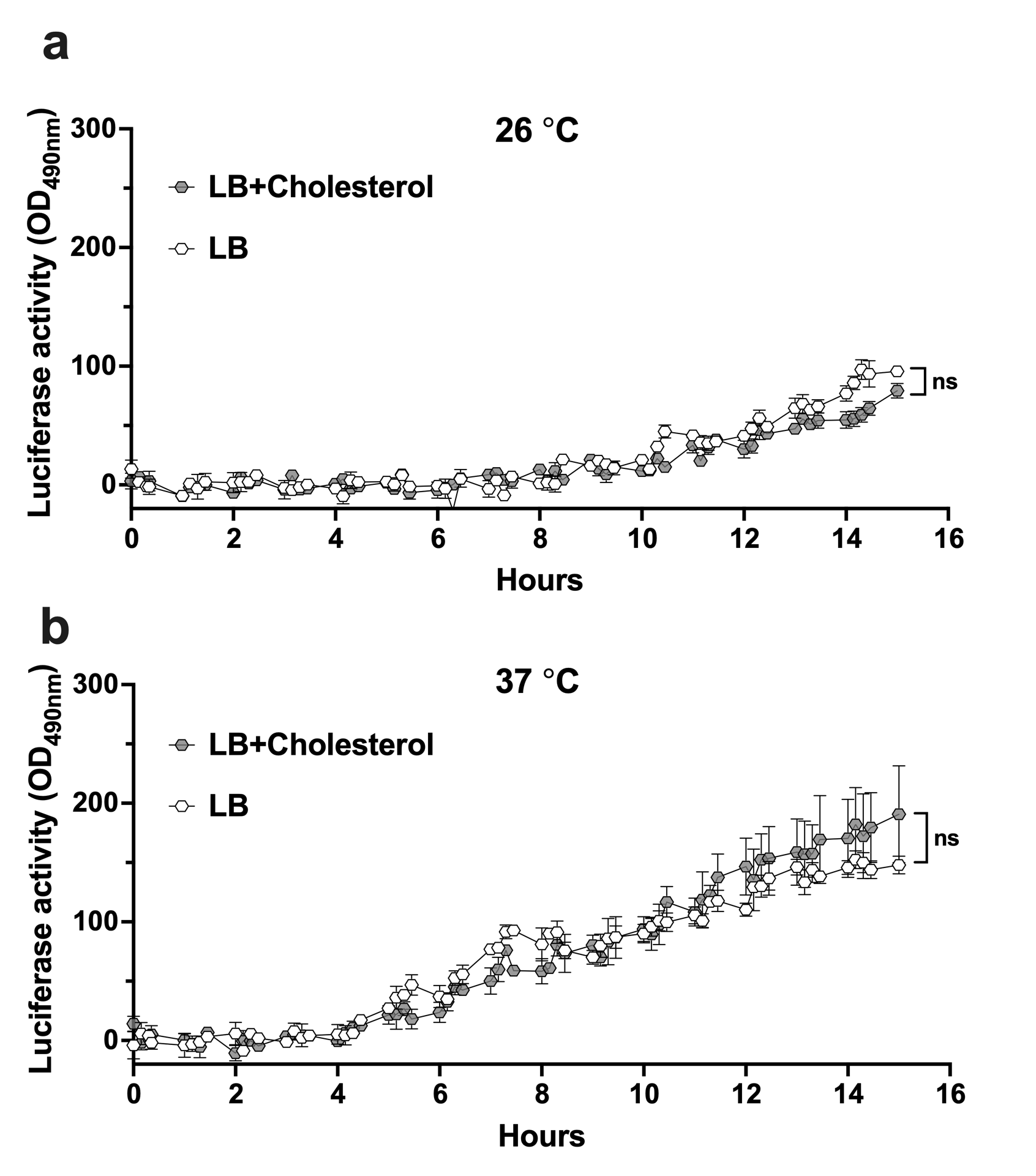

Supplement: Fig. S4 — Luciferase activity of the curli (csgDEFG) promoter. [file mbio.03242-24-s0004.tiff]
