# Supplementary material for: A temporary cholesterol-rich diet and bacterial extracellular matrix factors favor Salmonella spp. biofilm formation in the cecum
Source: mBio. 2024 Dec 5;16(1):e03242-24. doi: 10.1128/mbio.03242-24 (PMC11708031; doi:10.1128/mbio.03242-24)
Supplement: Legends — Supplemental figure legends. [file mbio.03242-24-s0005.docx]

**Supplementary material.**

**Supplementary Figure 1**. **a)** Bacterial growth curves of *Salmonella* cultured in TSB medium over a 15-hour period, with readings taken every 15 minutes (OD_600_). The strains used are indicated. **b)** Quantification of the area under the curve (AUC) from the growth curves in (a). Statistical analysis was performed using two-way ANOVA followed by Fisher’s LSD test.

**Supplementary Figure 2**. **a)** Gastrointestinal tract colonization was assessed by quantifying CFUs of freshly collected fecal samples. Mice fed with either a Ld or a Nd were subsequently infected with *S.* Tm ECM single mutants each lacking curli (*ΔcsgA*), colanic acid (*ΔwcaM*), O antigen capsule (*ΔyihO*) or cellulose (*ΔbcsE*) genes or a quadruple mutant lacking in all the above (ECM*^mut^*). Days are indicated. CFUs of homogenized tissue were isolated from infected mice with either **b)** single or **c)** quadruple ECM mutant strain at the indicated DPI. Mesenteric lymph nodes (MLN); small intestine (SI) and large intestine (LI). Dotted line represents ± quartiles. A minimum of three mice per condition were utilized for these analyses. Statistical analysis was conducted using Two-Way ANOVA followed by Fisher’s LSD test. *p<0.05, **p<0.01, ***p<0.001 and ****p<0.0001.

**Supplementary Figure 3. a)** Methodology schematic (detailed in the methods section). Samples were isolated from various tissues or feces to optimize the isolation of bacteria, cholesterol measurement, and flow cytometry. **b)** Quantification by serial dilution of cholesterol isolated from the cecum of infected mice at 21 DPI. **c)** Quantification of distribution of *S.* Tm in the cecum samples from infected mice using IHC. Primary Ab *S.* Tm LPS was employed. Strains and dietary conditions are indicated. **d)** IF image showing the visualization of biofilms in the cecum from mice infected with *S.* Tm*^wt^* at 7 DPI. Asterisks represent the lumen of the cecum. The primary Abs ⍺-amyloid proteins (red, curli), ⍺-CSA-1 (yellow, *S.* Tm), ⍺-Villin 1 (green, a cecal epithelium marker) and Fluorescent Brightener 28 (blue, cellulose) were used. Optical magnification 60X, scale bar: 5μm. **e)** Dot plots of colonies displaying *S.* Typhi morphology were analyzed by flow cytometry. Colonies with different characteristics were used as a negative control. The morphology of the colonies is shown in the dot plots. The primary Abs ⍺-*S.* Typhi CSA-1 and ⍺-*S.* Typhi LPS were used. **f)** Microscopic examination (H&E) was analyzed to determinate anatomic changes at 7 in the cecum of mice infected with *S.* Typhi*^wt^*. Optical magnification 40X, scale bar: 50 μm. At least four mice per condition were utilized. Error bars represent ± SD. Statistical analysis was conducted using Two-Way ANOVA followed by Fisher’s LSD test. *p<0.05, **p<0.01, ***p<0.001 and ****p<0.0001

**Supplementary Figure 4**. Luciferase activity of the curli (*csgDEFG*) promoter *S*. Tm (*S.* Tm*^lux:csgDEFG^*) cultured in LB medium over a 15-hour period with readings taken every 15 minutes (OD_490_). LB+Cholesterol represents cultures grown on cholesterol-coated plates. Cultures grown under **a)** ambient or **b)** physiological temperatures. Statistical analysis of AUC was performed using two-way ANOVA followed by Fisher’s LSD test.
